# Dietary quality and its structural relationships among equivalent income, emotional well-being, and a five-year subjective health in Japanese middle-aged urban dwellers

**DOI:** 10.1186/s13690-015-0081-0

**Published:** 2015-07-16

**Authors:** Sayuri Kodama, Nobuya Fujii, Tadashi Furuhata, Naoko Sakurai, Yoshinori Fujiwara, Tanji Hoshi

**Affiliations:** 1 Department of Health and Nutrition, School of Human Ecology, Wayo Women’s University, Chiba, Japan; 2 Graduate School of Urban System Science, Tokyo Metropolitan University, Tokyo, Japan; 3 Graduate School of Medicine, The Jikei University, Tokyo, Japan; 4 Tokyo Metropolitan Institute of Gerontology, Tokyo, Japan

**Keywords:** Dietary quality, Equivalent income, Emotional well-being, Subjective health, Middle-age

## Abstract

**Background:**

Although dietary quality in middle-age and the prime age of a person’s work career might be determined by positive emotional well-being based on socioeconomic status (SES), causation among determinants of dietary quality still remains unclear. Our purpose was to elucidate the structural relationships among five-year prior dietary quality, equivalent income, emotional well-being, and a five-year subjective health by sex and age group separately.

**Methods:**

In 2003, 10,000 middle-aged urban dwellers aged 40-64 years, who lived in ward A in the Tokyo metropolitan area, were randomly selected and a questionnaire survey was conducted by mail. In 2008, we made a follow-up survey for dwellers, and were able to gather their survival status. A total of 2507, middle-aged men (n = 1112) and women (n = 1395), were examined at baseline. We created three latent variables for a structural equation modeling (SEM*),* five-year subjective health reported in 2003 and in 2008, dietary quality of principle food groups diversity and eating behavior in 2003, and emotional well-being constructed by enjoyment & *ikigai* (meaning of life) and by close people in 2003. Equivalent income in 2003 was calculated as SES indicator.

**Results:**

In the SEM analysis of both men and women, there was an indirect effect of the equivalent income on dietary quality and on five-year subjective health, via emotional well-being explained by *ikigai* and having comforting people close to the individuals, significantly. There tended to be a larger direct effect of emotional well-being on the dietary quality in men than in women, and also a larger effect accompanying with aging. In women, there was a large direct effect of equivalent income on dietary quality than in men. When examined comprehensively, there appeared to be a larger effect of five-year prior equivalent income on subjective health during five-year in men than in women.

**Conclusion:**

This study suggests that it is necessary to support the improvement of dietary quality in middle age by considering the characteristics of sex and age group and also by providing supportive environment to enhance emotional well-being based on equivalent income, cooperating different field professionals to provide such as employment or community support program.

## Background

Japan has the longest healthy life expectancy (HLE) for both men and women among 187 major countries in the world [1]. However, there is about a one decade gap between HLE and life expectancy (LE) involving care needs’ period. In the 21st century, developed countries have become longevity societies. To maintain the quality of life in old age, living by themselves without care needs would be important. Moreover, in order to decrease national nursing-care cost, extension of HLE is crucial. To achieve closing the gap between HLE and LE, we should strengthen a supportive system of cooperation of professionals from different fields. Not only for elderly people but also for middle-aged people, whose lifestyles might be established, creating supportive environment for extending HLE might be needed and useful.

One background factor that Japan has the longest HLE could be improved dietary quality (DQ), which results in overall improved health factors, subsequent extending HLE. The improvement in DQ includes the establishment of a dietary environment that enables desirable healthy eating behavior and intake of diverse foods. Such improved DQ is speculated to have contributed to disease prevention and decreased mortality risk. DQ is a term that reflects a concept of desirable diet, used widely for assessing relationship with cancer or disease risk [2]. Even if a lot of indicators have developed for DQ, there has not been an established consensus of definition. Nowadays, dietary patterns have been used as an indicator of DQ in epidemiology study, rather than single nutrients or food groups [3–6]. Zazpe, et al. [4] reported that in 16,000 middle-aged Spanish adults, 47 % reduction of all-cause mortality risk was found in the highest adherence to the Mediterranean dietary patterns’ group consuming rich vegetables, fruits, fish, and olive oil. DASH (Dietary Approaches to Stop Hypertension) dietary pattern, which is rich in fruits, vegetables, and low-fat foods, reduced blood pressure increasing with age and risk for stroke in a Chinese population [5]. In Japan, diversity food pattern called “Japanese diet” characterized by rice staple, diverse plant origin foods, i.e. vegetables and soy, and fish intake, might be contributed to extend our HLE [6], yet scientific evidence for the associations have not been fully established. Especially, a causal relationship between DQ and HLE including the surrounding factors still remains unclear internationally.

HLE has been measured by two kinds of indicators. One is disability-free LE [1,7], and the other is subjective health (SH) i.e. self-rated health [8,9]. SH has been reported as a predictor of subsequent diseases, all-cause mortality [10–15], and as an indicator related to elder people’s attitude toward aging [16]. From a systematic review of 22 cohort studies, persons reporting “poor” SH had almost two-hold higher mortality risk compared with persons reporting “excellent” SH [14]. Even if a lot of studies about relationships between SH and mortality, more efforts need to elucidate the relationships between SH and surrounding factors. Especially, the relationships between DQ and SH, which are both independently predictors of mortality [17], have not been fully elucidated.

It is highly likely that various confounding factors are involved in the strong relationship between diet and health-related risks. It has become common knowledge in the public health field that socioeconomic status (SES), such as annual income and educational attainment, can determine between DQ and health risk [18]. Mejean et al. [19] followed up approximately 33,000 male and female in middle-age in the Netherlands for 12 years. Individuals with low education and employment status had increased incidence and mortality risk for cardiovascular disease and stroke. From this increase, dietary factors were the most significant intermediary determinants independent of sex and age. McLeod et al. [20] examined the indirect effects of nutritional knowledge on eating behavior. Path analysis revealed that the relationship between maternal education level and nutritional knowledge had an indirect effect on DQ. Zarnowiecki et al. [21] reported that the indirect determinants of children’s nutritional knowledge were parental dietary knowledge and the dietary environment around schools.

Many Japanese studies on diet and SES to date have been cross-sectional surveys on risk analysis adjusted for multiple factors [22,23]. However, the direct relationship of DQ with disease and mortality rate is insufficient to establish a causal relationship. Thus, structural elucidation is necessary, including underlying background factors, by conducting a longitudinal survey following subjects’ change. Hoshi et al. [24] examined the structural relationship between dietary habits and HLE in elderly subjects by tracking the subjects, same as the baseline’s subjects, and their survival every three years for six years. They indicated that SES, such as annual income and educational attainment, are likely the confounding factors in the relationship between dietary habits and HLE. Fujii et al. [25] examined the causal effect relationship, and showed that dietary habits of healthy elderly subjects were determined by three health-rated dimensions (physical, mental, and social health status) based on SES using a six years follow-up data.

Hoshi [26] showed that dietary and lifestyle habits in the elderly might be determined by SES-based health-related dimensions of three previous years via factors of enjoyment and *ikigai* (meaning of life). This result suggests that DQ might be a resultant factor determined by positive emotional health related to SES: hereinafter operationally defined as emotional well-being (EWB). Some studies have distinguished EWB from life satisfaction in the evaluation of life [27]. The relationship of EWB has been reported with mental health [28] and with survival, including prevention of stroke in the elderly [29–32]. Ostir et al. [29] conducted a six-year prospective cohort study and found that positive affect was inversely associated with the incidence of stroke in the elderly. Seale et al. [30] reported that positive emotion was associated with an increased level of recovery after stroke in the elderly. A nationally representative panel study was conducted in the U.S. with approximately 50,000 elderly subjects. It showed that elderly subjects with greater purpose of life had a lower incidence of stroke even after adjustment for SES [32].

*Ikigai* is a concept of EWB unique to Japan [33,34] and is considered to be extending beyond subjective well-being and extends into the future and in a social direction [33]. It has been reported that middle-aged individuals (40-79 years) with *ikigai* tended to have a reduced risk for mortality from stroke and cardiovascular disease [35,36]. There is a high level of stress, including from overworking, in middle-aged individuals (40-64 years) in the prime of their working life. It has been speculated that the following factors contribute greatly to stress management: EWB related to support of family and people around the individuals, and *ikigai* and enjoyment such as hobbies outside of work. DQ in middle age can also be a resultant factor determined by EWB based on the background factor SES. However, no study has been reported in Japan or overseas which examined the structural relationship between DQ in middle age and EWB. Since good dietary habits in middle age can determine HLE in old age [37], it is important not only to prevent early death but also to maintain DQ in middle age.

The present study aimed to elucidate the structural relationships among five-year prior DQ, equivalent income, EWB such as *ikigai*, and a five-year SH in Japanese middle-aged urban dwellers. The structural relationships were examined by sex and age group. The elucidation of structural relationships will enable multidisciplinary support, involving cooperation of professionals from different fields, to improve DQ in middle age. It is expected to result in effective dietary education, which in turn will lead to HLE in old age.

In this study, comprehensive evaluation of diet, showing a significant correlation with the five-year survival, was thought to reflect the qualitative condition of SES. Thus, the evaluation of both dietary content and eating behavior was operationally defined as the evaluation of “DQ” and used in the analysis.

## Methods

### Survey methods and participants

The participants of this study were middle-aged urban dwellers aged 40-64 years who lived in ward A in the Tokyo metropolitan area. Ward A was located north-east of Tokyo with a population of approximately 200,000. LE in the ward was shorter than in Tokyo. Especially, premature death rate of middle-aged men in the ward was higher than the national average. Moreover, taxable income per person was also low level compared with the other 23 wards in Tokyo.

A questionnaire was created which included SES factors and lifestyle factors, including dietary habits predicted to have a relationship with extension of HLE and prevention of early death. In 2003, 10,000 middle-aged people (40-64 years old) living in ward A were randomly sampled. A self-administered questionnaire was sent by postal mail and subsequently collected. A similar questionnaire survey was conducted 5 years later to track their survival status (living or deceased). By 2008, a total of 4443 (44.4 % response rate), middle-aged men (n = 2058, 46.3 %) and women (n = 2385, 53.7 %) were able to follow up. Deaths were confirmed in a total of 73 subjects (1.6 %), consisting of 57 men (2.8 %) and 16 women (0.7 %), and their survival times in days were determined. We excluded 654 participants who did not respond for 12 choices annual income question (n = 195, 4.4 %) or who chose answer of “I do not want to disclose my income information” (n = 459, 10.8 % of all participants, men: 8.9 % of all men, women: 12.5 % of all women) in the first survey. A total of 2507, middle-aged men (n = 1112, 44.4 %) and women (n = 1395, 55.6 %) of survivors, who responded in the second survey in 2008, were examined at baseline in the present study (Table 1).

**Table 1:** Characteristics of participants

			Men							Women						
			40 ~ 49 years		50 ~ 59 years		60 ~ 64 years		*p*	40 ~ 49 years		50 ~ 59 years		60 ~ 64 years		*p*
			n = 298 (26.8 %)		n = 505 (45.4 %)		n = 309 (27.8 %)			n = 373 (26.7 %)		n = 611 (43.8 %)		n = 411 (29.5 %)		
Socioeconomic status	(SES)															
Equivalent income (03EI)	<1 million yen	n (%)	35	11.7 %	69	13.7 %	62	20.1 %	***	78	20.9 %	143	23.4 %	136	33.1 %	***
	1million-3million yen		94	31.5 %	164	32.5 %	157	50.8 %		125	33.5 %	234	38.3 %	180	43.8 %	
	3million-5million yen		116	38.9 %	147	29.1 %	52	16.8 %		105	28.2 %	140	22.9 %	67	16.3 %	
	5million-7million yen		50	16.8 %	99	19.6 %	23	7.4 %		61	16.4 %	79	12.9 %	18	4.4 %	
	>7million yen		3	1.0 %	26	5.1 %	15	4.9 %		4	1.1 %	15	2.5 %	10	2.4 %	
Family structure	Single	n (%)	40	13.5 %	58	11.5 %	37	12.1 %	***	22	5.9 %	49	8.0 %	54	13.1 %	***
	Husband and wife		25	8.4 %	86	17.0 %	83	27.0 %		27	7.2 %	106	17.4 %	127	30.9 %	
	Parents-and-children		123	41.4 %	192	38.0 %	92	30.0 %		174	46.6 %	243	39.8 %	122	29.7 %	
	Others		109	36.7 %	169	33.5 %	95	30.9 %		150	40.2 %	212	34.8 %	108	26.3 %	
Employment patterns	Full‐time	n (%)	261	87.6 %	431	85.9 %	194	63.0 %	***	165	44.4 %	255	41.9 %	118	28.7 %	***
	Part-time		21	7.0 %	44	8.8 %	54	17.5 %		128	34.4 %	208	34.2 %	126	30.7 %	
	Full‐time housewife		0	0 %	0	0 %	0	0 %		68	18.3 %	125	20.5 %	128	31.1 %	
	Out of employment		16	5.4 %	27	5.4 %	60	19.5 %		11	3.0 %	21	3.4 %	39	9.5 %	
Dietary quality	(03DQ)															
Dietary content	PFDS	mean ± SD*	18.4 ± 3.3	(10.0-28.0)	17.6 ± 3.4	(8.0-26.0)	17.5 ± 3.4	(10.0-26.0)	**	20.0 ± 3.4	(10.0-28.0)	19.3 ± 3.3	(10.0-26.0)	19.1 ± 3.4	(9.0-26.0)	***
Eating behavior	Eating behavior score	mean ± SD*	4.9 ± 1.4	(0.0-8.0)	5.1 ± 1.4	(1.0-8.0)	5.3 ± 1.2	(1.0-8.0)	***	6.2 ± 1.3	(1.0-8.0)	6.2 ± 1.2	(2.0-8.0)	6.3 ± 1.2	(1.0-8.0)	n.s
	Desirable eating behavior		2.2 ± 1.2	(0.0-5.0)	2.4 ± 1.2	(0.0-5.0)	2.6 ± 1.0	(0.0-5.0)	***	3.4 ± 1.1	(0.0-5.0)	3.5 ± 1.0	(0.0-5.0)	3.5 ± 1.0	(0.0-5.0)	n.s
	Undesirable eating behavior		2.7 ± 0.6	(0.0-3.0)	2.7 ± 0.6	(0.0-3.0)	2.8 ± 0.6	(0.0-3.0)	n.s	2.8 ± 0.5	(0.0-3.0)	2.8 ± 0.5	(0.0-3.0)	2.8 ± 0.5	(0.0-3.0)	n.s
Emotional well-being	(03EWB)															
Enjoyment & *ikigai*	0	n (%)	22	7.4 %	44	8.7 %	17	5.5 %	n.s	13	3.5 %	22	3.6 %	13	3.2 %	n.s
	1		39	13.1 %	68	13.5 %	42	13.6 %		33	8.8 %	51	8.3 %	33	8.0 %	
	2		74	24.8 %	109	21.6 %	79	25.6 %		70	18.8 %	118	19.3 %	70	17.0 %	
	3		153	51.3 %	270	53.5 %	160	51.8 %		230	61.7 %	383	62.7 %	281	68.4 %	
	4		5	1.7 %	12	2.4 %	10	3.2 %		19	5.1 %	27	4.4 %	9	2.2 %	
	5		3	1.0 %	1	0.2 %	1	0.32 %		7	1.9 %	9	1.5 %	5	1.2 %	
	6		2	0.7 %	1	0.2 %	0	0 %		1	0.3 %	1	0.2 %	0	0 %	
	Enjoyment & *ikigai* score	mean ± SD*	2.3 ± 1.0	(0.0-6.0)	2.3 ± 1.0	(0.0-6.0)	2.3 ± 1.0	(0.0-5.0)	n.s	2.6 ± 0.9	(0.0-6.0)	2.6 ± 0.9	(0.0-6.0)	2.6 ± 0.8	(0.0-5.0)	n.s
Close people	Many close people	n (%)	30	10.1 %	45	9.0 %	23	7.5 %	n.s	44	11.9 %	54	8.9 %	31	7.5 %	n.s
	Several close people		160	53.7 %	277	55.3 %	162	52.9 %		247	66.6 %	425	69.8 %	283	68.9 %	
	Almost no close person		78	26.2 %	147	29.3 %	85	27.8 %		71	19.1 %	100	16.4 %	76	18.5 %	
	No close person		30	10.1 %	32	6.4 %	36	11.8 %		9	2.4 %	30	4.9 %	21	5.1 %	
	Close people score	mean ± SD*	2.6 ± 0.8	(1.0-4.0)	2.7 ± 0.7	(1.0-4.0)	2.6 ± 0.8	(1.0-4.0)	n.s	2.9 ± 0.6	(1.0-4.0)	2.8 ± 0.6	(1.0-4.0)	2.8 ± 0.6	(1.0-4.0)	n.s
Five-year subjective	health (5yrSH)															
Subjective health (SH) in 2003	Healthy	n (%)	93	31.4 %	163	32.5 %	96	31.7 %	n.s	123	33.0 %	177	29.2 %	118	29.1 %	n.s
	Somewhat healthy		163	55.1 %	274	54.7 %	178	58.7 %		207	55.5 %	358	59.0 %	226	55.8 %	
	Somewhat unhealthy		29	9.8 %	46	9.2 %	23	7.6 %		35	9.4 %	55	9.1 %	37	9.1 %	
	Unhealthy		11	3.7 %	18	3.6 %	6	2.0 %		8	2.1 %	17	2.8 %	24	5.9 %	
Subjective health (SH) in 2008	Healthy	n (%)	86	29.1 %	146	29.1 %	107	34.7 %	n.s	99	27.0 %	149	24.5 %	112	27.3 %	n.s
	Somewhat healthy		170	57.4 %	284	56.7 %	169	54.9 %		212	57.9 %	383	63.0 %	241	58.8 %	
	Somewhat unhealthy		34	11.5 %	48	9.6 %	26	8.4 %		48	13.1 %	62	10.2 %	38	9.3 %	
	Unhealthy		6	2.0 %	23	4.6 %	6	1.9 %		7	1.9 %	14	2.3 %	19	4.6 %	

Items of enjoyment & *ikigai*: Exercise and sports, hobby, trip, working or study, relaxing with family, children’s growth, communicating with friends, activities in area or with another groups

*PFDS* Principle food groups diversity score Chi-square test, *n.s* non-significant. * mean ± SD* (Minimum - Maximum) of scores. ** *p* < 0.01. *** *p* < 0.001

A survey committee was established in ward A to conduct the surveys. The study was confirmed to comply with the confidentiality obligations under the Public Service Act and used only identification numbers as personal codes. The study was performed with the approval of the ethics committee of the Graduate School of Urban System Science at Tokyo Metropolitan University.

### Survey items

This study created a scoring system as following to make variables of examination for the structural relationship between baseline data in 2003 and follow-up one in 2008 in the middle-aged urban dwellers (Table 1).

At the baseline, the subjects were asked about the amount of their annual income, SES such as family structure and employment patterns. Based on a previous study [38], our study used equivalent income (EI) which was calculated by dividing the median value of the multiple-choice annual income by the square root of the household size. We used the EI as observational variable reflecting SES in the analysis.

For the items of dietary content diversity, this study used, the “principle food groups diversity score” (PFDS) reported to have a relationship with the cumulative survival rate in a previous study [39] by the authors of this study. There were six food groups that had a significant positive relationship with the survival time of baseline subjects: fresh milk and milk products, green vegetables, yellow vegetables, potatoes, fresh fruits, and meats. The scores were totaled for their frequencies of intake (6-30 points), and the sum was established as the PFDS which evaluates the qualitative aspect of the diet (Table 2).

**Table 2:** Correlation analysis between five-year survival time and dietary factors

	Correlation coefficients	Points
Dietary content		
Fresh milk and milk products	0.056^**^	5point: Almost everyday
Green vegetables	0.045^**^	4point: 5 ~ 6 days/week
Yellow vegetables	0.045^**^	3point: 3 ~ 4 days/week
Potatoes	0.039^**^	2point: 1 ~ 2 days/week
Fresh fruits	0.039^**^	1point: Almost not eating
Meats	0.032^*^	
Principle food groups diversity score	0.060^***^	6 ~ 30point
Seaweeds	0.030^n.s^	
Soybean products	0.027^n.s^	
Fresh fish and shellfish	0.009^n.s^	Out of score
Small fish	0.006^n.s^	
Eggs	−0.017^n.s^	
Eating behavior		
Desirable eating behavior		
Preparing a meal by myself	0.048^**^	
Obtaining diet and nutrition information	0.039^*^	1point: Yes
Consuming diversity foods	0.038^*^	0point:No
Having a meal at a fixed time	0.036^*^	
Using supplement frequently	0.033^*^	
Undesirable eating behavior		
A lot of food likes and dislikes	−0.076^**^	1point: No
Skipping a meal frequently	−0.060^**^	0point: Yes
Having a poor appetite frequently	−0.048^**^	
Eating behavior score	0.065^**^	0 ~ 8point
Eating between meals frequently	0.026^n.s^	
Trying to losing weight	0.020^n.s^	
Knowing optimal dietary content and quantity	0.013^n.s^	
Eating outside frequently	−0.029^n.s^	Out of score
Using cooked and instant food frequently	−0.026^n.s^	
Taking enough time for a meal	−0.007^n.s^	
Having optimal dietary quantity	−0.005^n.s^	

Kendall’s tau rank correlation analysis * *p* < 0.05, ** *p* < 0.01, *** *p* < 0.001, *n.s* non-significant

Cronbach’s coefficient α: Principle food groups diversity score (0.727), Eating behavior score (0.470)

**Fig. 1:**
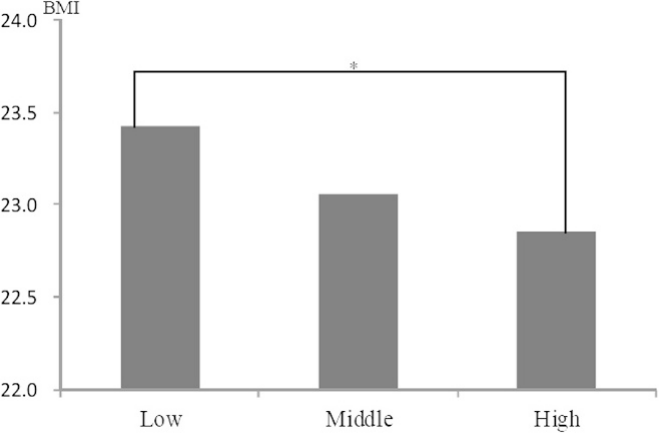
Comparison between three categories of the principal food groups diversity score and BMI

To test if excessive energy intake would be a confounding factor for the PFDS, the participants of this study were divided into 3 categories by the PFDS, and we compared each category’s average body mass index (BMI), which were calculated by the subjects’ self-reported weight and height (Fig. 1). BMI was used as an indicator reflecting energy balance based on intake and consumed energy. It was observed that the highest PFDS category’s participants’ average BMI were smaller than those of the lowest one, significantly. These results showed that excessive energy intake might not be confounding factor for the dietary diversity.

For eating behavior, the items used were those related to the evaluation of eating behavior involving 15 food types. Scoring was similar to that for the dietary content and was performed on questions that showed a significant relationship with the survival time of baseline subjects. A score of 1 point was given if the answer was “yes” to a question with a positive relationship with the survival time, and a score of 0 points was given if the answer was “no”. The behavior related to these questions was considered “desirable eating behavior”. A score of 1 point was given if the answer was “no” to a question with a negative relationship with the survival time, and a score of 0 points was given if the answer was “yes”. The behavior related to these questions was considered “undesirable eating behavior”. The eating behavior score was calculated using the sum (0-8 points) of scores for desirable eating behavior and undesirable eating behavior.

For EWB at baseline, we made an indicator of enjoyment & *ikigai*. The question was “What do you enjoy most in life & *ikigai*?” The participants answered more than one choice from nine items. A score of 0 point was given if they chose “nothing special” to the question. Each 1 point was given for the chosen item. The sum of this question’s point was considered as an indicator of “enjoyment & *ikigai*”. For the other EWB reflecting social support, we made an indicator showing the number of comforting people close to the individuals. The question was “Do you have comforting people close to you?” A score of 4 point was given if the participants answered “Yes, I have many close people”, and a score of 3 points for “several close people”, 2 points for “almost no close person”, 1 point for “no close person”.

The question about the SH in baseline (2003) and five years later (2008) were “How do you feel about your present health condition?” A score of 4 points was given for “healthy”, 3 points for “somewhat healthy”, 2 points for “somewhat unhealthy”, and 1 point for “unhealthy”.

### Relationship between survey items’ scores and cumulative survival rate

**Fig. 2:**
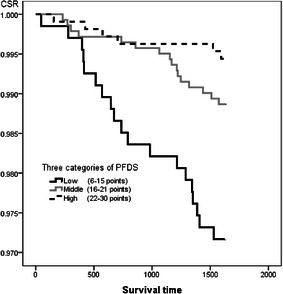
The comparison of cumulative survival rate among principal food groups diversity score in three categories

**Fig. 3:**
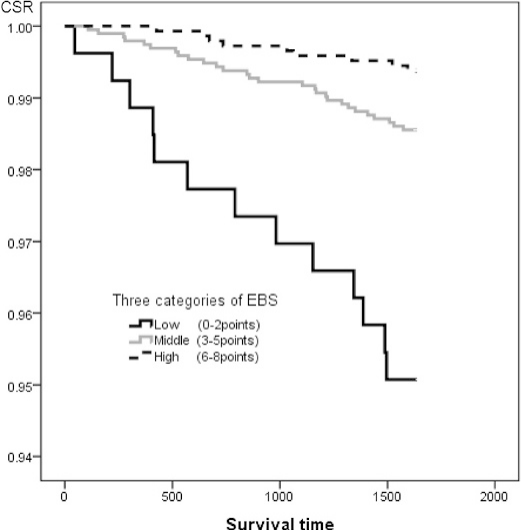
The comparison of cumulative survival rate among eating behavior score in three categories

Each survey items were divided into 3 or 4 categories by its scores, and we examined if these survey items would be an adequate index for predicting a five-year survival, comparing their cumulative survival rate of five years. For the dietary diversity and eating behavior score, the cumulative survival rate of five years in the high and middle categories were significantly maintained high rate comparing with the lowest one (Log-rank, *p* < 0.001) (Fig. 2, 3).

**Fig. 4:**
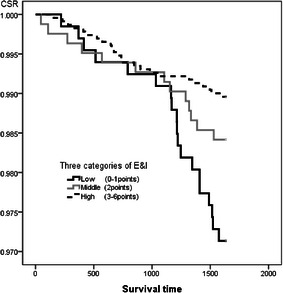
The comparison of cumulative survival rate among enjoyment & ikigai in three categories

**Fig. 5:**
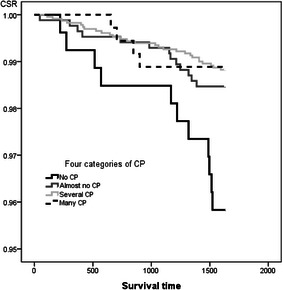
The comparison of cumulative survival rate among close people score in four categories

For the enjoyment & *ikigai* score, the cumulative survival rate of five years in the highest category was significantly maintained high rate comparing with the lowest one (Log-rank, *p* < 0.01) (Fig. 4). For the close people, the cumulative survival rate of five years in the third categories; several, were significantly maintained highest rate comparing with the lowest one (Log-rank, *p* < 0.01). The category of having many close people was slightly lower rate than the third categories of having several close people (Log-rank, *p* < 0.01) (Fig. 5).

Thus, we made clear that each survey items might be an adequate index for predicting a five-year survival, and were able to use as indicators examining the present study’s aim, elucidating the structural relationships among SH related to the premature death rate of middle-age.

### Exploratory factor analysis for the survey items

To create latent variables for SEM, we conducted exploratory factor analysis (Table 3). The survey items examined by the factor analysis were 7 items. SH in 2003 and in 2008, PFDS, eating behavior score, enjoyment & *ikigai*, comforting people, and EI in 2003. We labeled factor 1 as “five-year subjective health (5yrSH)” (“” means latent variable) constructed by the 2003 and 2008 SH. PFDS reflecting the score of dietary diversity, and eating behavior score in factor 2 was labeled as “03 dietary quality (03DQ)”, and enjoyment & *ikigai* and close people in factor 3 was as “03 emotional well-being (03EWB)”. The EI in 2003 (03EI), obtaining from factor 4, was used as a basis of the structural relationship in a hypothesized model.

**Table 3:** Results of exploratory factor analysis for the survey items

	Factor loading			
	Factor1	Factor2	Factor3	Factor4
03 subjective health	0.746	0.014	−0.014	0.060
08 subjective health	0.739	−0.021	0.009	0.050
03 PFDS	−0.016	0.754	−0.039	0.279
03 eating behavior score	0.016	0.531	0.082	−0.268
03 enjoyment & *ikigai* score	−0.036	−0.010	0.750	0.160
03 close people score	0.034	0.005	0.501	0.068
03 equivalent income	0.083	0.046	0.144	0.198
Cumulative contribution ratio	20.8 %	32.9 %	38.4 %	40.7 %
Cronbach’s coefficient α	0.999	0.390	0.484	-

Maximum likelihood estimation and Promax oblique rotation were performed

*03* 2003 years, *08* 2008 years

### Analysis method

Kendall rank-order analysis was used to determine the relationship between the survival time and the variables constructing for the “03DQ”. Structural equation modeling (SEM) was used for evaluating the goodness of being fit of the hypothesized model of the structural relationships based on SES among DQ, EI, EWB, and 5yrSH at the five-year follow-up. The analysis software used was SPSS 21.0, AMOS 21.0 for Windows, and the level of statistical significance was set at p < 0.05.

## Results

### Comparison of each item by sex and age group

The properties of the surveyed items are shown by sex and age group in Table 1. Examination was performed on the percentage of men with 03EI (reflecting SES) of less than 3,000,000 yen. The percentage tended to increase significantly in men beyond the retirement age in their 60s (70.9 %) compared with men in their 40s (43.2 %) and 50s (46.2 %). The percentage also tended to increase significantly in women in their 60s (76.9 %) compared with women in their 40s (54.4 %) and 50s (61.7 %).

In the family structure of the present participants, the percentage of single family rate in men was almost stable, as the age group became older (40s: 13.5 %, 50s: 11.5 %, 60s: 12.1 %). While, the percentage of single family rate in women tended to increase significantly, as the age group became older (40s: 5.9 %, 50s: 8.0 %, 60s: 13.1 %).

For “03DQ”, the frequency of diverse intake of principle food groups tended to significantly decrease as the age group became older in both men and women. In men, desirable eating behavior tended to significantly increase as the age group became older. In women, no change in eating behavior was observed with age group.

When examination was performed on the number of items of enjoyment and *ikigai* (composing “03EWB”), the percentage of individuals choosing 2 or 3 items was 80 % overall in all age groups. No significant difference was observed by sex or age group. The percentage of individuals choosing 4 or 5 items tended to increase as the age group became older in men, but the percentage tended to decrease with age in women. Examination was performed on EWB related to the number of comforting family members or friends who were people close to the individuals, and no significant difference was observed by sex or age group. However, there was a higher percentage of women (77.9 %) than men (63.1 %) who answered that there were “several close people” or “many close people”. In men and women, fewer individuals tended to answer “many close people” and more individuals tended to answer “no close person” as the age group became older.

When examination was performed on SH in 2003 using “5yrSH”, the following percentages of men answered that they were “healthy” or “somewhat healthy” showed almost unchangeable, as the age group became older. In women, the percentage tended to decrease, albeit not significantly, as the age group became older: 88.5 % in their 40s, 88.2 % in their 50s, and 84.9 % in their 60s. There was a similar tendency in 2008. There was a significantly positive correlation between SH in 2003 and that in 2008 (men: r = 0.456 and women: r = 0.520), and SH tended to be stable even five years later.

### Structural relationships among dietary quality, equivalent income, and a five-year subjective health

**Fig. 6:**
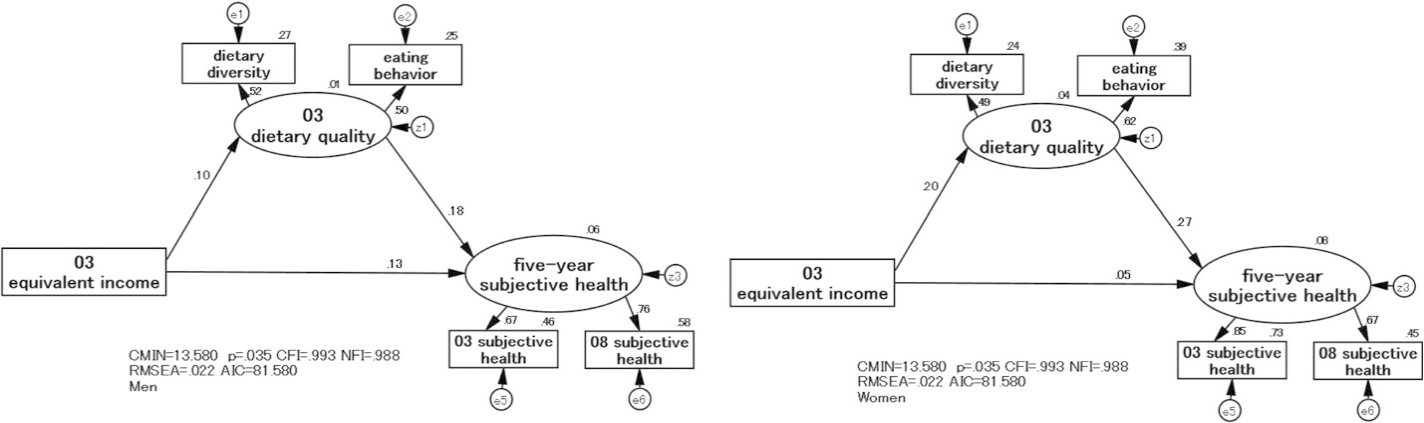
Structural relationships among “03 dietary quality”, 03 equivalent income, and “five-year subjective health” in men (left) and women (right)

First, a model was established which excluded “03EWB”, and examination was performed on whether “03DQ” of middle-aged urban dwellers could be an intermediary determinant of 03EI and “5 years SH” (Fig. 6 and Table 4). This model was created based on 03EI and used the “hypothesized conceptual model of health disparities” [40] as a reference which was presented in a previous ecological study by the authors of this study.

**Table 4:** Standardized effects of the SEM models by sex

Standardized effects	Men	Women
Direct effect		
03EI → 03DQ	0.103	0.197
03EI → 5yrSH	0.133	0.054
03DQ → 5yrSH	0.183	0.267
Indirect effect		
03EI → 03DQ → 5yrSH	0.019	0.052
Comprehensive effect		
03EI → 03DQ → 5yrSH	0.151	0.106

*EI* equivalent income, *DQ* dietary quality, *5yrSH* five-year subjective health, *03* 2003 years, *08* 2008 years

In a model for men, the standardized estimate was 0.133 for a direct effect of 03EI on “5yrSH”. The standardized estimate was very low at 0.019 for an effect of 03EI which indirectly determined “5yrSH” via “03DQ”. In a model for women, there was a small direct effect of 03EI on “5yrSH” (0.054), and their relationship was not statistically significant. There was a direct effect of 03EI on “03DQ” (0.197) and a direct effect of “03DQ” on “5yrSH” (0.267), and the indirect effect was larger in women (0.052) than in men (0.019). The above results showed that “03DQ” of female middle-aged urban dwellers was more prone to be determined by 03EI than that of their male counterparts. The results suggest that “03DQ” could be an intermediary determinant of 03EI and “5yrSH”.

The model fit indices were CFI = 0.993, NFI = 0.988, and RMSEA = 0.022, indicating a good fit. However, the coefficient of determination of “5yrSH” was small in both men and women.

### Structural relationships among dietary quality, equivalent income, emotional well-being, and a five-year subjective health

Next, the hypothesized model, which included “03EWB”, was established using as a reference the model of Hoshi [26] on dietary and lifestyle habits, enjoyment, and *ikigai* in elderly individuals. Examination was then performed on which factor, “03DQ” or “03EWB”, has a larger effect as an intermediary determinant of 03EI and “5yrSH” in middle-aged urban dwellers. In addition, analysis was performed on the comprehensive causal structure for “5yrSH” based on 03EI.

**Fig. 7:**
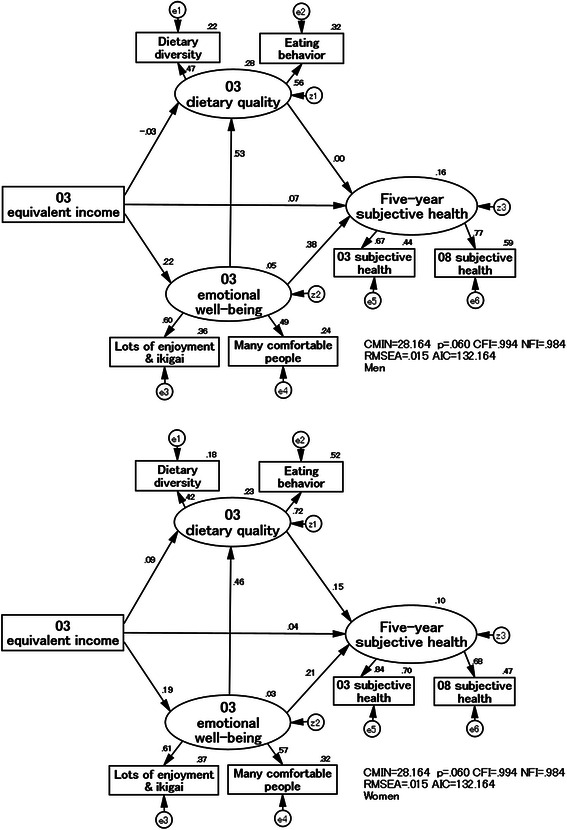
Structural relationships among “03 dietary quality”, 03 equivalent income, “03 emotional well-being”, and “five-year subjective health” in men (upper) and women (lower)

Figure 7 and Table 5 show the results of simultaneous analysis by men and women. In the model for men, the standardized estimate was smaller, albeit not significantly, for the direct effect of 03EI on “03DQ”. On the other hand, the standardized estimate was 0.224 for the direct effect on “03EWB”. The standardized estimate was large at 0.533 for the direct effect of “03EWB” on “03DQ”. There was a greater indirect effect on “03DQ” via “03EWB” (0.119) compared with a direct effect from 03EI. The standardized estimate was largest for the direct effect of “03EWB” (0.378) on “5yrSH”. The above results showed that “03EWB” tended to have a larger effect than “03DQ” as an intermediary determinant of 03EI and “5yrSH” in male middle-aged urban dwellers. “03DQ” was largely determined by “03EWB”, which in turn was determined by SES.

**Table 5:** Standardized effects of the SEM models by sex and age group separately

	Men				Women			
Standardized effects	40 ~ 49 years	50 ~ 59 years	60 ~ 64 years	All age^a^	40 ~ 49 years	50 ~ 59 years	60 ~ 64 years	All age^a^
Direct effect								
03EI → 03EWB	0.081	0.264	0.284	0.224	0.196	0.127	0.272	0.187
03EI → 03DQ	−0.039	−0.063	0.034	−0.028	0.096	0.151	−0.047	0.085
03EI → 5yrSH	0.212	0.010	0.095	0.066	0.019	−0.036	0.122	0.043
03EWB → 03DQ	0.299	0.488	0.802	0.533	0.333	0.409	0.580	0.461
03EWB → 5yrSH	0.314	0.461	0.482	0.378	0.249	0.055	0.349	0.205
03DQ → 5yrSH	0.039	−0.009	−0.213	−0.005	0.131	0.237	0.047	0.155
Indirect effect								
03EI → 03EWB → 03DQ	0.024	0.129	0.228	0.119	0.065	0.052	0.158	0.086
03EWB → 03DQ → 5yrSH	0.012	−0.004	−0.170	−0.003	0.044	0.097	0.027	0.071
03EI → 03EWB → 03DQ → 5yrSH	0.025	0.121	0.082	0.084	0.070	0.055	0.100	0.084
Comprehensive effect								
03EI → 03DQ	−0.015	0.066	0.261	0.091	0.161	0.203	0.111	0.171
03EWB → 03DQ → 5yrSH	0.325	0.457	0.312	0.375	0.293	0.151	0.376	0.276
03EI → 03EWB → 03DQ → 5yrSH	0.237	0.131	0.177	0.150	0.089	0.019	0.222	0.107

Analyses by sex and age group simultaneously:

CMIN = 51.899, p = 0.556, CFI = 1.000, NFI = 0.971, RMSEA = 0.000, AIC = 363.899

^a^Analyses by sex simultaneously:

CMIN = 28.164, p = 0.060, CFI = 0.994, NFI = 0.984, RMSEA = 0.015, AIC = 132.164

*EI* equivalent income, *DQ* dietary quality, *EWB* emotional well-being, *5yrSH* five-year subjective health, *03* 2003 years, *08* 2008 years

In the model for women, the standardized estimate was larger (0.085) for the direct effect of 03EI on “03DQ” compared with the model for men. However, it was smaller than the direct effect on “03EWB” at 0.187. The standardized estimate was 0.461 for the direct effect of “03EWB” on “03DQ”. The level was similar for the effect of 03EI that directly determined “03DQ” and its indirect effect (0.086) via “03EWB”. The standardized estimate was largest for the effect of “03EWB” (0.205) on “5yrSH”, and the direct effect of “03DQ” (0.155) was larger than in men. In female middle-aged urban dwellers, there tended to be a larger mediating effect of “03EWB” and “03DQ” as intermediary determinants of 03EI and “5yrSH”.

When the results were examined comprehensively, men, more than women, tended to show a larger effect on “5yrSH”, which was based on 03EI of SES (men: 0.150 and women: 0.107). The model fit indices were CFI = 0.994, NFI = 0.984, and RMSEA = 0.015, indicating a good fit. The coefficient of determination was 16 % in men and 10 % in women for “5yrSH”, and the explanatory power was increased by the addition of “03EWB” to the models.

The subjects were divided into 6 groups by sex and age group, and simultaneous analysis was performed (Table 5). When the relationship was examined between 03EI and “03DQ”, there was a larger indirect effect (0.024-0.228) via “03EWB” compared with a direct effect (-0.039 - 0.034) in all age groups in men. In women, the direct effect tended to be larger in their 50s (0.151) and the indirect effect tended to be larger in their 40s (0.065) and 60s (0.158). The direct effect (0.096) was also observed in women in their 40s but was not significant. The direct effect of “03EWB” on “03DQ” tended to become larger as the age group became older in both men and women. In particular, the standardized estimate was large at 0.802 for men in their 60s. When examination was performed on the comprehensive effect of 03EI of SES on “5yrSH”, the effect tended to be large in men in their 40s (0.237) and women in their 60s (0.222).

The model fit indices were CFI = 1.000, NFI = 0.971, and RMSEA = 0.000, indicating a good fit. The coefficient of determination was 14-21 % in the men’s model and 7-18 % in the women’s model for “5yrSH”.

## Discussion

### Structural relationships among dietary quality, equivalent income, emotional well-being, and a five-year subjective health

The novel aspects of this study are that the findings of the five-year follow-up were reflected in the SH of middle-aged urban dwellers and that sex and age group differences were elucidated in the structural relationship among DQ, EI, EWB explained by *ikigai* and having reliable family members or friends, i.e. social support, and a five-year SH. In our SEM model (Fig. 7), we explored that for the effect from five-year prior EI on DQ, the indirect effect (simple regression from EI to EWB and from EWB to DQ) was larger than the direct effect (simple regression from EI to DQ). For the effect from five-year prior EI on five-year SH, the indirect effect (simple regression from EI to EWB, from EWB to DQ, and from DQ to five-year SH) was larger than the direct effect (simple regression from EI to five-year SH). That is, EWB mediated the relationship among EI, DQ, and a five-year SH in both men and women. In addition, in women’s model, not only EWB but also DQ mediated the relationship between EI and a five-year SH. These results suggest that providing supportive environment to enhance EWB based on EI might be useful for improving DQ in middle-aged urban dwellers.

*ikigai* is a concept that is unique to Japan and was defined operationally as EWB in this study. It is considered to be a concept that extends beyond subjective well-being and extends into the future and in a social direction [33,34]. EWB is independent of mental health and is considered to be one of the factors associated with health [27,28]. The results of this study suggest that it will be necessary to consider the interrelationship with EWB: *ikigai* and social support, in dietary education support which leads to improved SH in middle age.

Comparing two types of results in our SEM analysis, in the level of direct effect of EI and five-year SH examined in the men’s model without EWB (Fig. 6), there could have been a spurious relationship via EWB. The significance was shown of SEM which can structurally analyze the related factors. In the women’s model, both DQ and EWB also affected five-year SH, and the significance of SEM was shown.

### Relationship between dietary quality and emotional well-being in middle-aged urban dwellers

Other novel aspects of this study are that the DQ was comprehensively evaluated using diverse dietary content and eating behavior shown to be associated significantly with the five-year cumulative survival rate and with significant negative collation with the average BMI. In previous studies, the SES-related DQ had often been evaluated by the consumption of good quality food which contributes to disease prevention [41]. Such food included fruit, vegetables, and low-fat food. However, these studies did not necessarily elucidate the relationship between SES and comprehensive evaluation of DQ, including of eating behavior. In addition, there have been only a small number of studies on the relationship between DQ and positive EWB. To our knowledge, our study is the first one that has focused on *ikigai* of EWB in middle age and has elucidated its structural relationship with DQ using a longitudinal study examining sex and age differences.

In our study, the analysis models showed that DQ tended to be determined largely by positive EWB in middle-aged urban dwellers. Hoshi [26] examined the relationship of *ikigai* and dietary habit in the elderly, and our results support the possibility that a similar relationship exists in middle-aged individuals. Hingle et al. [42] compared the most optimistic and the least optimistic menopausal women. In the most optimistic women, positive EWB was positively correlated with approximately threefold improvement in DQ one year after dietary intervention compared with the least optimistic women. This result is consistent with ours.

When our examination focused on sex and age group differences, men had a higher standard estimate than women for a direct effect of EWB on DQ and on a five-year SH. In particular, men in their 60s showed a direct effect of EWB on DQ that was approximately 1.4 times than in women in their 60s. Conklin et al. [43] examined social relationships and healthful dietary behavior in middle-aged individuals in the UK. They reported that men living alone or with infrequent friend contact tended to have a markedly lower DQ with a small variety of fruits and vegetables compared with women. That is, the DQ in men seemed to be greatly affected by social factors such as marital status and contact with friends. From the above results, it is speculated that support for improved DQ in middle-aged men is necessary and effective in conjunction with support for EWB based on the background factor SES.

In women, EI reflecting SES showed a large direct effect on DQ. This result supported the models in previous studies of Hoshi et al. [24] and Fujii et al. [25] with the elderly as subjects. Thus, generalizability was increased with applicability in different generations. The direct effect of SES on diet was greater in women than in men because women are responsible for the household budget in many cases in Japan. Thus, it was speculated that DQ in women tended to be determined more likely by SES of the household [44].

### Support for improving dietary quality to improve subjective health of middle-aged urban dwellers

In middle-aged urban dwellers, the results of this study suggest the need for not only support for dietary content and eating behavior in dietary education but also for positive EWB explained by *ikigai* and social support based on SES. In particular, the need for such support was shown in high age groups’ middle-aged men. About the family structure of men in the present study, rate of living alone, which was reported as low DQ [43], was the highest in their 40s. While, the employment patterns’ change related to aging was observed notably. The rate of part-time employment in their 60s was approximately 1.5 times, and out of employment rate was about 4 times than those of in their 40s. That is, in the middle-aged men of urban dwellers of present study, low SES, i.e. low income related to the employment patterns’ change, seems to affect strongly to the direct effect of EWB on DQ, which became larger accompanying with aging. In the future, the number of women in the work force will further increase, and the relationship with social factors will likely increase. Thus, both men and women need increased dietary educational activities and support for EWB based on SES.

About the family structure of women in the present study, rate of living alone was highest in their 60s. It was two times over than that of women in their 40s. The following points can be likely to be given as one of reasons. As we mentioned in the methods, premature death rate of middle-aged men in the ward A was higher than in national average. Moreover, the employment pattern’s change related to aging in women was observed smaller than in men. Especially, part-time employment rate in women’s all age groups was kept approximately 30 %. That is, in the middle-aged women of urban dwellers in the present study, SES related to the family structure’s change (living alone) seems to affect strongly to the direct effect of EWB on DQ, which became larger accompanying with aging. For creating supportive environment for the women, program related to community support might be effective for enhancing their EWB, which results in DQ improvement.

EWB does not always increase in all socioeconomic levels when SES represented by income is increased. Kahneman et al. [27] showed that high income was positively correlated with a higher life satisfaction level, and there was a steady rise in life satisfaction with income over the entire income range. In contrast, there was improvement in EWB only up to a certain income level ($75,000). Low income groups had increased divorce and health problems, and the life satisfaction level and EWB tended to decrease. Toyokawa et al. [45] reported that access to healthcare tended to be limited in low income groups due to financial reasons and speculated that the impact on mental health and EWB differs by SES level. It is necessary to accumulate further evidence because detailed measures by SES level are needed to devise effective support.

### Research topics

First, we used SEM for evaluating the structural relationships based on SES among DQ, EI, EWB, and five-year SH with a longitudinal study. More effort would be needed to establish the causality confirmed reproducibility. In our new interventional research, we would like to compare targeted groups with the control groups.

Moreover, when the coefficient of determination was examined for the structural relationship among DQ, EI, EWB, and five-year SH, the coefficient determining five-year SH was 16 % in men and 10 % in women. Thus, the explanatory power was not strong. In future studies, it will be necessary to elucidate the causal structure with DQ by examining not only the positive aspects of EWB but also negative indices such as stress related to SES. Many previous studies have analyzed the risk factors, including depressive symptoms [46], and negative affect [47,48], to examine the relationship of diet based on SES with mental health and EWB. Miyaki et al. [22] indicated that low folic acid intake, income, and educational history could be related to depressive symptoms in middle-aged workers. Hayman et al. [48] examined low income women and reported that indifference toward diet was more common than emotional- and stress-eating, which are risk factors for obesity. Hiyoshi et al. [49] used the results of secondary analysis of the Comprehensive Survey of Living Conditions in Japan and reported a relationship of SES with psychological factors due to stress.

Second, in our study, dietary diversity, which composes DQ, was scored using frequency of food intake. Thus, food and nutrients were not quantified. A further topic of research is the examination of the validity of DQ evaluation in quantitative evaluation. Previous studies have used the SES indices of income, just as in our study, in addition to educational history [18–22,24–26,41,47,48,50]. Since educational history is an index that shows high sensitivity in the relationship with health disparity, and DQ in children, the next generation might be determined by their mother’s education levels [50], it is a factor that we would like to examine in our future studies. Moreover, our present study did not ask the subjects about the exact annual income and instead asked a multiple choice question with 12 choices of the range of income. However, women tended to be more reluctant to disclose their income information. This study aimed to elucidate the structural relationship based on SES, and these subjects had to be excluded in the analysis despite providing valuable information for other items. Another future topic of research is the promotion of research in Japan by increasing awareness that SES is important in improving the standard of health in the overall local region.

## Conclusion

This study examined survival in middle-aged urban dwellers using a baseline survey and a follow-up survey conducted five years later. In both men and women, SES due to five years prior EI tended to indirectly determine the DQ and a five-year SH via EWB. The results of our study suggest that it is necessary to support the improvement of DQ in middle age by considering the characteristics by sex and age group and also by providing supportive environment to enhance EWB based on EI. Creating action with cooperating different field professionals to provide such as employment or community support program for retired men and for women living alone might be beneficial for improvement of DQ.
